# Ultrasound-Guided Physiotherapy for Symptomatic Ulnar Nerve Dislocation in a Collegiate Baseball Pitcher: A Case Report

**DOI:** 10.7759/cureus.95903

**Published:** 2025-11-01

**Authors:** Hiroyuki Kobayashi, Masashi Kawabata, Koji Wagatsuma, Tomoki Miwa, Wataru Iwamoto

**Affiliations:** 1 Department of Rehabilitation, Medical Base Shinkoiwa, Tokyo, JPN; 2 Department of Rehabilitation, Kitasato University School of Allied Health Sciences, Sagamihara, JPN; 3 Department of Rehabilitation, Jinseikai, Tokyo, JPN; 4 Department of Sports Medicine, Edogawa Hospital, Tokyo, JPN

**Keywords:** cubital tunnel, dynamic ultrasound, myofascial trilaminar retinaculum, neurogenic thoracic outlet syndrome, return to play, throwing athlete, ulnar nerve dislocation

## Abstract

Symptomatic ulnar nerve (UN) dislocation is an under-recognized cause of medial elbow pain in overhead athletes. Traditionally, surgery has been considered when symptoms persist; however, advances in dynamic ultrasound (US) and US-guided targeted interventions may enable conservative treatment. Reports on long-term return to play (RTP) after US-guided physiotherapy remain scarce.

A 20-year-old right-handed collegiate sidearm pitcher developed progressive medial elbow pain for one year, with acute worsening two weeks before presentation. Clinical examination suggested ulnar collateral ligament (UCL) strain and concomitant neurogenic thoracic outlet syndrome (N-TOS). Dynamic US revealed an anterior UN dislocation over the medial epicondyle during elbow flexion, which reproduced the patient’s pain.

Management was performed using a stepwise strategy. Phase 1 targeted the proximal contributors (N-TOS) using scapular and chin retraction exercises combined with neural mobilization. Phase 2 addressed the primary distal pathology with US-guided perineural soft tissue mobilization, and skin-pinch traction was applied until movement of the myofascial trilaminar retinaculum (MTR) was confirmed under US, thereby improving the perineural tissue. This was followed by pain-free UN-sliding exercises.

After 12 weeks, neural tension signs subsided, and pain during dynamic UN displacement diminished. A graded throwing program was completed, enabling RTP at five months. At the two-year follow-up, the athlete remained asymptomatic without recurrence, despite persistent painless dynamic subluxation on US.

In this single-case report of an athlete with UN dislocation complicated by N-TOS, a stepwise, ultrasound-guided, target-specific physiotherapy approach is presented as a hypothesis-generating example of conservative management. The intervention, which included direct mobilization of the myofascial trilaminar retinaculum, facilitated a safe return to play, and no surgical intervention was required during the treatment course. In this case, ultrasound was used provisionally to monitor clinically relevant changes, such as visible improvement in neural gliding and reduction of perineural soft tissue irritation. Rather than serving as a generalized recommendation, these observations suggest a potential rationale for incorporating ultrasound into conservative management pathways.

## Introduction

Symptomatic anterior dislocation of the ulnar nerve (UN) at the elbow is increasingly recognized in overhead athletes, although reported prevalence figures predominantly reflect dynamic findings on ultrasonography or physical examination rather than clinically manifest disease. Dynamic instability has been observed in up to 44% of young baseball players, 88% of collegiate athletes, and 38.5% of professional players [1-3]. Such instability can provoke medial elbow pain, paresthesia, and weakness, particularly during the acceleration phase of throwing, thereby impairing athletic performance [4]. In routine clinical practice, initial management typically involves rest, bracing, activity modification, or general physiotherapy, with surgery reserved for persistent or progressive symptoms. Anterior transposition has traditionally been considered the primary surgical option when conservative care fails; however, it carries well-documented risks such as recurrence, neuritis, and delayed return to play (RTP), with reported RTP rates of approximately 70%-80% and recovery times averaging 10-12 months [5]. Conversely, nonspecific conservative strategies may offer only partial or temporary relief, and no standardized nonoperative pathway currently exists. Surgery remains the preferred option in cases of pronounced instability or when structured conservative interventions do not achieve meaningful improvement.

Recent advances in high-resolution dynamic ultrasound (US) have enabled direct visualization of UN kinematics and surrounding perineural tissues, facilitating the accurate identification of symptomatic dislocations and real-time, targeted intervention [6]. Importantly, dynamic US also allows clinicians to distinguish true dislocation from subluxation, terms that are often used interchangeably in the literature but indicate different degrees of ulnar nerve instability. From a pathophysiological standpoint, symptom severity may be influenced by both distal nerve irritation and proximal neural compromise. A representative example is neurogenic thoracic outlet syndrome (N-TOS), characterized by brachial plexus compression at the thoracic outlet. In throwing athletes, repetitive overhead motion and scapular dysfunction can predispose to N-TOS and exacerbate UN-related symptoms through a "double-crush" mechanism [7].

To the best of our knowledge, no previous reports have described sustained RTP in a competitive pitcher with concomitant UN dislocation and N-TOS managed exclusively with a structured, ultrasound-guided physiotherapy program. In this context, particular attention was given to the myofascial trilaminar retinaculum (MTR), whose close anatomical relationship with the ulnar nerve renders it susceptible to adhesions that restrict neural gliding. What distinguishes the present case is the incorporation of a stepwise, ultrasound-guided MTR mobilization strategy while concurrently addressing proximal contributors such as N-TOS. Our aim is to demonstrate how dynamic US can serve not only as a diagnostic tool to visualize nerve displacement but also as a means of guiding targeted intervention within a conservative management framework for complex peripheral nerve conditions in throwing athletes.

## Case presentation

A 20-year-old male collegiate pitcher competing in the Division I League baseball presented with a one-year history of progressive, medial right elbow pain. He was a right-handed thrower (height: 175 cm, weight: 72 kg) with no prior elbow surgery or systemic diseases. The patient primarily played as a relief pitcher, averaging 20-30 pitches per appearance, twice per week, during the competitive season.

Initially, pain occurred only after practice but gradually progressed to constant discomfort associated with paresthesia radiating to the ring and little fingers. Over the preceding three months, he continued to pitch while taking oral analgesics. Two weeks before presentation, the pain and numbness markedly worsened, leading to an inability to throw competitively and to prompt medical evaluation.

On initial examination, the clinical findings suggested an ulnar collateral ligament (UCL) strain and concomitant N-TOS. Complementary magnetic resonance imaging (MRI) findings supported the classification of the UCL injury as a grade I sprain. However, the mild nature of this sprain did not account for the patient’s neuropathic symptoms or dynamic pain reproduction during elbow flexion and forearm rotation, making ulnar nerve pathology the primary focus of management. The side-to-side difference in valgus laxity on dynamic ultrasound was minimal, indicating no clinically significant instability. High-resolution dynamic ultrasound (US; Hitachi Aloka Arietta Prologue, L64 linear probe, 18-5 MHz; frequency: 6.67 MHz, depth: 2.7 cm, gain: G69, frame rate: 36 fps) was performed with the patient in the supine position, shoulder and elbow flexed at 90°. The ulnar nerve (UN) was imaged in the short axis at the cubital tunnel. This confirmed abnormal anterior translation of the UN across the medial epicondyle, reproducing the patient’s characteristic symptoms.

Initial clinical findings

During shadow pitching at maximal external rotation, the patient reported severe medial elbow pain, rated 9 out of 10 on the Numerical Rating Scale (NRS). Physical examination revealed nearly full shoulder motion (flexion: 175°, abduction: 170°) but a painful limitation of external rotation (90° versus 100° on the contralateral side). At the elbow, flexion was restricted to 110° because of pain, whereas extension was full. Rotator cuff strength was preserved with the arm at the side for external and internal rotation; however, teres minor strength assessed at 90° of shoulder abduction was a manual muscle testing (MMT) grade 4/5. Grip strength was markedly diminished on the dominant side (22 kg versus 49 kg on the contralateral side).

Provocative and neurological tests

To standardize interpretation and improve reproducibility, positive criteria were defined as follows: Roos test, symptom onset within 30 seconds; Adson and Wright tests, pain reproduction accompanied by attenuation of the radial pulse; ULNT (ulnar nerve-biased), performed with shoulder abduction/external rotation, scapular depression, elbow flexion, forearm supination, and wrist/finger extension (positive if symptoms occur during elbow flexion); and Tinel’s sign, reproduction of paresthesia upon tapping at ≥1 of the evaluated sites (arcade of Struthers, cubital tunnel, and Osborne ligament).

Cervical root involvement was considered unlikely because the Jackson compression test and Spurling test were negative. However, thoracic outlet-related maneuvers were positive, the Roos test (elevated arm stress test) reproduced medial elbow pain within 15 seconds, and both the Adson test (cervical rotation with deep inspiration while monitoring radial pulse) and the Wright test (hyperabduction maneuver with radial pulse assessment) were also positive, accompanied by tenderness of the scalene and pectoralis minor muscles. Palpation revealed focal tenderness along the course of the ulnar nerve at the medial elbow, particularly around the cubital tunnel. Neurodynamic assessment showed a strongly positive UN-biased upper limb neurodynamic test (ULNT). Multiple sites elicited a positive Tinel’s sign, including the arcade of Struthers, cubital tunnel, and Osborne ligament. Additionally, mild weakness and atrophy of the abductor digiti minimi were observed, which was consistent with ulnar neuropathy.

Ultrasound assessment

**Figure 1:**
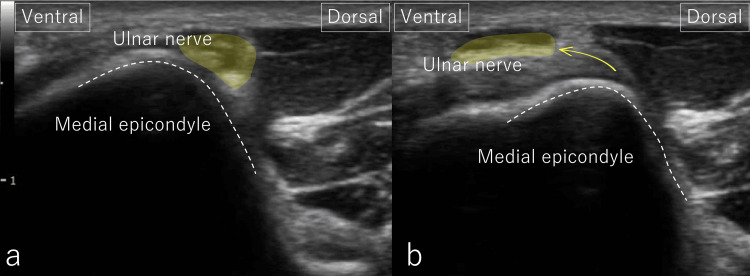
Dynamic ultrasound findings at initial presentation (short-axis view) At the cubital tunnel, the UN was dislocated over the medial epicondyle. (a) With the elbow flexed at 60°, the UN was located posterior to the medial epicondyle. (b) At 90° flexion, the UN translated anteriorly, reproducing the patient’s pain. Both images were obtained with the forearm in supination. UN: ulnar nerve

High-resolution dynamic US was performed using a linear high-frequency probe along the short axis of the cubital tunnel. Gravity stress US demonstrated a side-to-side valgus joint line gapping difference of only 0.4 mm, indicating the absence of clinically meaningful UCL laxity. Although dynamic US-based criteria for pathological ulnar nerve displacement have been proposed, no universally accepted cutoff values are currently established. Accordingly, in this case, the degree of displacement was evaluated in relation to symptom reproduction and classified as grade 3 (complete dislocation) according to the intraoperative system described by Mirza et al. [8]. This grading was applied by an experienced musculoskeletal sonographer using the intraoperative criteria as a reference standard to improve reproducibility. Although no precise millimetric cutoff has been established, the displaced ulnar nerve visibly translated beyond the medial epicondylar ridge, and its anterior excursion consistently reproduced the patient’s characteristic symptoms. The displacement pattern was reproducible across repeated elbow flexion-extension cycles and was assessed by an experienced musculoskeletal sonographer using Mirza et al.’s intraoperative criteria as a reference standard. With elbow flexion from 60° to 90°, the UN translated anteriorly over the medial epicondyle [9,10], reproducing the patient’s characteristic pain (Figure 1, Video 1). To clarify diagnostic reasoning, the initial differential diagnoses included UCL sprain, cubital tunnel syndrome, cervical radiculopathy (C8/T1), pronator syndrome, and neurogenic thoracic outlet syndrome (N-TOS). UCL injury was deprioritized due to minimal valgus laxity and MRI findings, cervical involvement was excluded by negative Spurling and Jackson tests, and pronator syndrome was ruled out based on the absence of proximal forearm tenderness. Persistent dynamic dislocation and Tinel positivity supported cubital tunnel pathology, with N-TOS considered a contributing proximal factor.

**Video 1:** Dynamic ultrasound before intervention During elbow motion from extension to flexion, the ulnar nerve dislocates over the medial epicondyle, causing pain. Measurement position: The patient was placed in the supine position with the shoulder and elbow flexed at 90°. The examination was performed in a short-axis view of the cubital tunnel. The motion was recorded from 60° to 90° of elbow flexion, with the forearm in supination.

Physiotherapy intervention

Management was implemented in a stepwise manner, addressing both proximal and distal contributors.

Phase 1 (Weeks 1-4: Proximal Factors, N-TOS)

Interventions aimed at improving the flexibility of the scalene and pectoralis minor muscles and correcting forward head posture [11,12]. The exercises included scapular retraction [11] and chin-retraction drills [12], combined with scapular elevation-depression movements. These were integrated with neural mobilization techniques to reduce mechanical stress on the brachial plexus [13].

Phase 2 (Weeks 5-12: Distal Factors, UN Dislocation)

**Figure 2:**
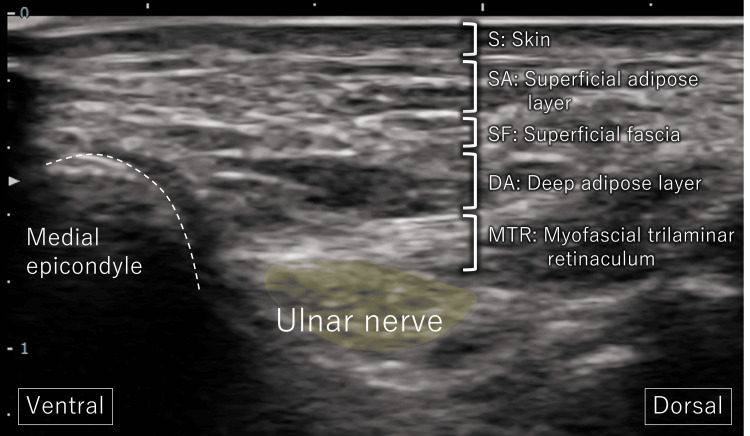
Anatomical depiction of the UN at the cubital tunnel Ultrasound depiction of perineural structures surrounding the UN, including the MTR. UN: ulnar nerve, MTR: myofascial trilaminar retinaculum, S: skin, SA: superficial adipose layer, SF: superficial fascia, DA: deep adipose layer

**Figure 3:**
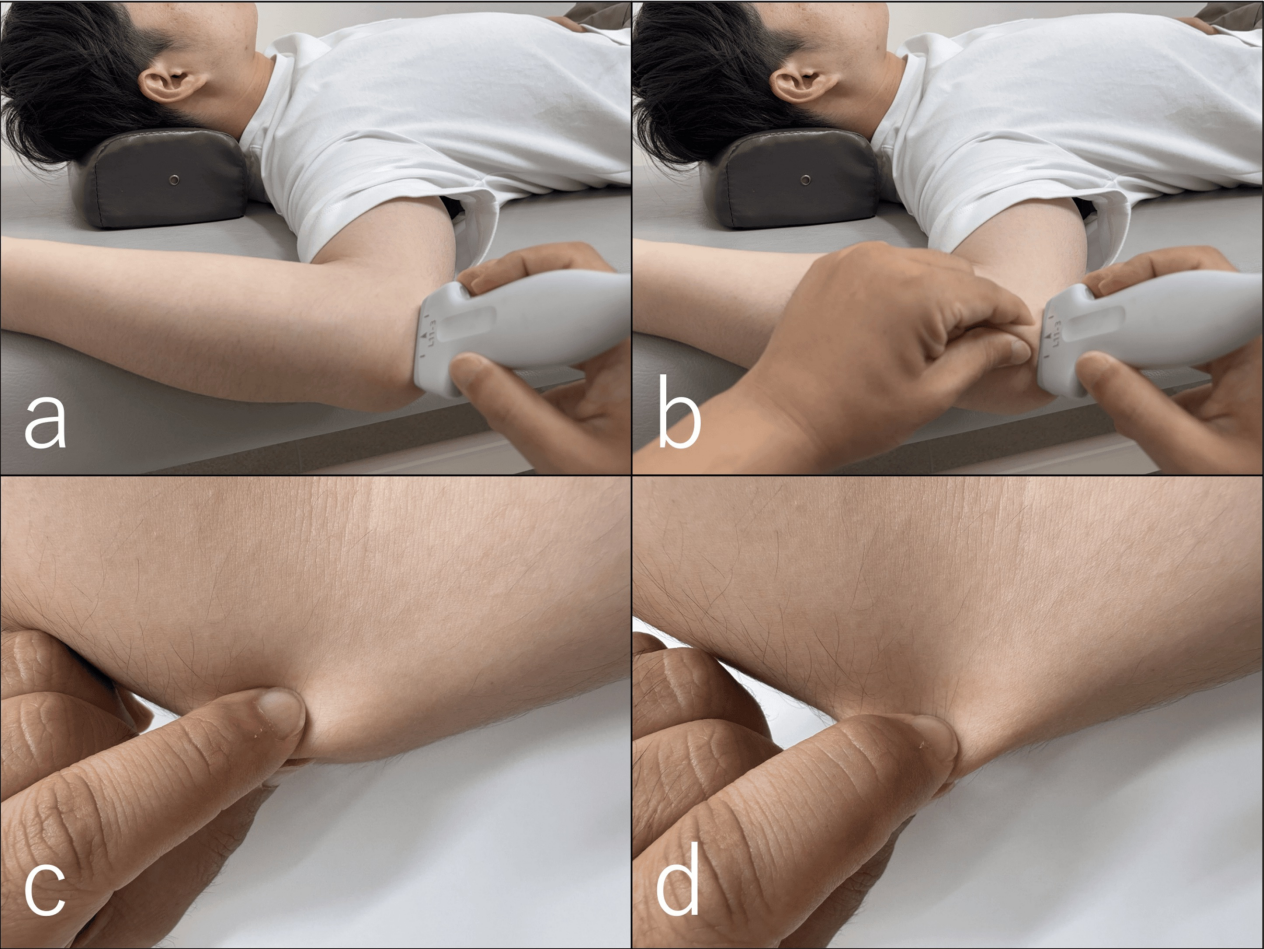
Ultrasound-guided skin-pinch traction technique at the cubital tunnel Under ultrasound guidance, the cubital tunnel was visualized, and soft tissue traction was applied until movement of the MTR was observed. (a) Probe position at the cubital tunnel. (b) In-plane overview of the maneuver. (c) Demonstration of skin pinch at the cubital tunnel without a probe for clarity. (d) Skin traction performed until the displacement of the MTR was achieved, illustrated without a probe for better visualization. MTR: myofascial trilaminar retinaculum

**Figure 4:**
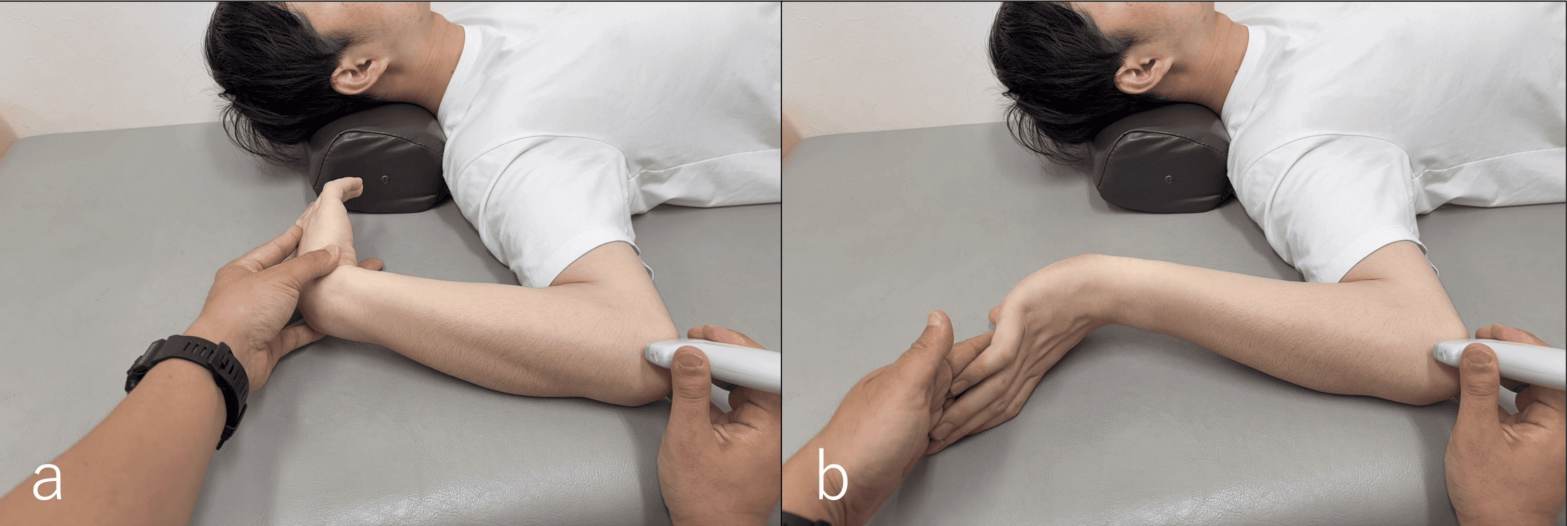
Ultrasound-guided UN sliding technique With the ulnar nerve imaged in the long axis at the cubital tunnel, sliding was induced in the pain-free range. (a) Wrist flexion with the elbow flexed and the forearm supinated. (b) Wrist extension with the elbow flexed and the forearm supinated. UN: ulnar nerve

The primary treatment target was the myofascial trilaminar retinaculum (MTR), defined as the three-layered myofascial complex forming the posteromedial roof of the cubital tunnel, superficial to the ulnar nerve and distinct from Osborne’s ligament (Figure 2) [14]. Under real-time ultrasound guidance, the MTR was visualized as a distinct hyperechoic retinacular structure superficial to the ulnar nerve, consistent with descriptions in prior sonographic studies [6,14]. Under ultrasound guidance, skin-pinch traction was applied at the sites with the strongest Tinel’s sign. Light-to-moderate vertical traction was used, sufficient to visually separate the MTR into its three hyperechoic layers on ultrasound imaging. Mobilization was confirmed when the MTR and adjacent perineural tissues were seen to glide relative to the ulnar nerve (Figure 3, Video 2). Each application was performed for approximately 5-10 seconds and repeated 5-10 times per session at the most Tinel-positive sites. The intervention was administered by a licensed physiotherapist with over 10 years of clinical experience in musculoskeletal practice. No adverse events, such as increased pain, swelling, or paresthesia, were observed during the sessions. Once the pain associated with the dislocation diminished, US-guided sliding techniques were initiated. With the shoulder abducted and externally rotated, the elbow was positioned in maximal flexion tolerated without symptoms [15], and the forearm was supinated [16]. Repetitive wrist flexion-extension was performed within a pain-free range to facilitate UN gliding (Figure 4, Video 3) [15,16]. For home exercises, both the self-applied skin-pinch traction maneuver and sliding technique were prescribed twice daily. Each session consisted of 5-10 repetitions per site, following the pain-free principle, and the patient was instructed to avoid symptom provocation. Progression was based on the resolution of pain and paresthesia, monitored weekly.

**Video 2:** Ultrasound-guided skin-pinch traction at the cubital tunnel Skin traction was applied until movement of the myofascial trilaminar retinaculum was confirmed to improve perineural mobility.

**Video 3:** Dynamic behavior of the ulnar nerve during the sliding technique The ulnar nerve was visualized at the cubital tunnel. With wrist dorsiflexion, the nerve slides distally, whereas with palmar flexion, it slides proximally.

Adherence and safety

The patient demonstrated high adherence to both supervised and home exercise programs, with a compliance rate exceeding 90%, confirmed by weekly logs and therapist interviews. Throughout the intervention period, no adverse events such as pain exacerbation, swelling, or neurological deterioration were observed.

Timeline and outcomes

RTP in this case was defined as pitching at the same pre-injury competitive level and completing at least one inning in official play without pain or symptom recurrence.

To clarify the temporal relationship between diagnostic findings, symptom changes, and targeted interventions, the clinical course was organized chronologically as follows.

Weeks 1-4

Weekly 40-minute sessions targeting N-TOS were conducted. Palpation-induced pain of the scalene and pectoralis minor muscles decreased, and the Roos, Adson, and Wright tests became negative. Grip strength improved markedly from 22 kg to 50 kg. However, ulnar nerve-related symptoms persisted, specifically residual Tinel’s signs and a positive ulnar nerve-biased ULNT.

Weeks 5-12

Weekly physiotherapy focused on UN mobility. Tinel’s sign and ULNT findings progressively improved, and abductor digiti minimi strength recovered. Dynamic US still demonstrated anterior subluxation of the UN; however, the pain associated with dislocation was reduced (Video 4).

**Video 4:** Dynamic ultrasound after intervention Although ulnar nerve dislocation persisted, gliding across the medial epicondyle became smoother, and pain was eliminated during flexion.

Weeks 13-20

Structured throwing progression was initiated, with gradual weekly increases in distance and intensity. During this partial return, intermittent symptom recurrences were managed by reapplying targeted interventions from phases 1 (for N-TOS) and 2 (for UN dislocation). Additionally, biomechanical corrections of faulty throwing mechanics have been incorporated to reduce medial elbow stress and optimize functional recovery.

Week 20

The patient successfully returned to competitive pitching without pain (NRS score: 0) and demonstrated improved range of motion, strength, and neurological function without recurrence of symptoms.

Long-Term Follow-Up

Despite persistent painless subluxation of the UN on dynamic US, the athlete remained symptom-free and continued to compete for two years without any recurrence (Table 1).

**Table 1:** Timeline of physiotherapy intervention and clinical course ROM: range of motion, p: pain at the medial elbow, →: continuation (no change in numerical value or continuation of intervention), ER at 90° ABD, shoulder external rotation at 90° abduction, MMT: Manual Muscle Testing, SSP: supraspinatus, ISP: infraspinatus, SSC: subscapularis, Tm: teres minor, FCU: flexor carpi ulnaris, ADM: abductor digiti minimi, UnAF/AF: unaffected side/affected side, NRS: Numeric Rating Scale, ST-arc: Struthers’ arcade, Cub-tun: cubital tunnel, Osb-lig: Osborne’s ligament, ULTT: upper limb tension test, -: no data, RTS: return to sport, N-TOS: neurogenic thoracic outlet syndrome, ulnar N: ulnar nerve

Parameter		Baseline (week 0)	Weeks 1-4	Weeks 5-8	Weeks 9-12	Weeks 13-20	Week 20
Elbow joint ROM (UnAF/AF)	Flexion	110°p/140°	120°p/140°	130°p/140°	140°p/140°	140°/140°	140°/140°
	Extension	0°/0°	0°/0°	0°/0°	0°/0°	0°/0°	0°/0°
Shoulder joint ROM (UnAF/AF)	Flexion	175°/180°	180°/180°	180°/180°	180°/180°	180°/180°	180°/180°
	Abduction	170°/180°	180°/180°	180°/180°	180°/180°	180°/180°	180°/180°
	ER at 90° ABD	90°p/110°	100°p/110°	110°p/110°	110°/110°	110°/110°	110°/110°
MMT (UnAF/AF) grade 0-5	SSP	5/5	5/5	5/5	5/5	5/5	5/5
	ISP	5/5	5/5	5/5	5/5	5/5	5/5
	SSC	5/5	5/5	5/5	5/5	5/5	5/5
	Tm	4/5	4/5	5/5	5/5	5/5	5/5
	FCU	4/5	4/5	4/5	5/5	5/5	5/5
	ADM	4/5	4/5	4/5	5/5	5/5	5/5
Grip strength	UnAF/AF	22/49 kg	50/49 kg	50/49 kg	50/49 kg	50/49 kg	50/49 kg
Tinel sign (NRS: /10)	ST-arc	6	2	1	0	0	0
	Cub-tun	8	5	3	2	1	0
	Osb-lig	7	3	1	1	0	0
Pain during ULTT (NRS: /10)	Ulnar	9	7	5	2	1	0
Playing level	-	No throw	→	Shadow pitching	15-30 m	Partial RTS	Full RTS
Treatment strategy	-	-	For N-TOS	For ulnar N	For N-TOS and ulnar N		
Physiotherapy intervention	-	-	Scapular retraction and chin retraction				
	-	-	-	Ulnar nerve grasping technique			
	-	-	-	-	Ulnar nerve sliding technique		

## Discussion

This case highlights the potential of US-guided physiotherapy as a nonsurgical option for symptomatic UN dislocation, a condition traditionally considered refractory to conservative treatment and frequently managed with anterior transposition surgery [5]. Despite the complexity of concomitant N-TOS [7], a stepwise approach addressing both proximal and distal factors enables full RTP.

US is particularly valuable for two reasons: First, it allowed for the precise identification of the most symptomatic sites, such as the strongest Tinel-positive regions, and enabled targeted intervention. Second, it provides real-time confirmation that the intended perineural tissues, including the MTR, are mobilized appropriately. The MTR, composed of deep fat, tendons, and muscle layers, is known to impair UN gliding when it is thickened or fused [14]. In this case, the direct mobilization of the MTR likely contributed to the improvement in neural dynamics. The immediate visualization of enhanced gliding and pain relief further underscores the unique advantages of US-guided manual techniques over conventional approaches.

Although neural sliding techniques are effective, excessive tensile stress may worsen symptoms in cases of marked entrapment or adhesion [17]. In the current case, prioritizing the treatment of the proximal contributor (N-TOS) before addressing the distal dislocation reduced the neural load and created conditions for the safe implementation of the sliding exercises. Thus, US guidance facilitated lesion targeting and provided reassurance of the safety and appropriateness of the intervention. From a clinical perspective, this report suggests that US-guided physiotherapy may be considered as a conservative option in selected athletes with symptomatic UN dislocation and serves as a hypothesis-generating rather than implying equivalence to surgery.

This study has several limitations that should be acknowledged. First, neither magnetic resonance imaging (MRI) nor electrodiagnostic testing (EDX) was performed. These modalities were not pursued because the patient’s symptoms and dynamic ultrasound findings were clinically concordant, and no red-flag signs suggesting alternative pathology were present. However, the absence of MRI and EDX may reduce diagnostic certainty, and such evaluations should be considered in cases with atypical symptoms, progressive deficits, or unclear localization to guide escalation of care. Second, the dynamic ultrasound assessment was conducted by an unblinded examiner, which may introduce measurement or observer bias. Third, this was a single-case report, limiting the generalizability of the findings. Accumulation of additional cases is needed to further clarify the potential effectiveness of this approach. Fourth, validated patient-reported outcome (PRO) measures, such as the Disabilities of the Arm, Shoulder and Hand (DASH) questionnaire or Kerlan-Jobe Orthopaedic Clinic (KJOC) score, were not collected. Future investigations should incorporate standardized PROs and objective benchmarks to strengthen external validity.

## Conclusions

Despite these limitations, in a collegiate baseball pitcher with symptomatic UN dislocation, a stepwise, US-guided physiotherapy program integrating proximal and distal interventions was associated with complete symptom resolution and successful return to competitive play.

This single case supports the feasibility of a conservative, US-guided rehabilitation strategy and suggests that it may serve as a hypothesis-generating treatment option in selected cases, emphasizing its potential for individualized management of UN instability. Future studies with larger cohorts, quantitative US outcome measures, validated patient-reported metrics, and predefined return-to-play criteria are warranted to determine the generalizability and comparative effectiveness of this approach.
